# Management der isolierten Amyloidose der Harnblase

**DOI:** 10.1007/s00120-025-02621-6

**Published:** 2025-06-12

**Authors:** Agneta Seebold, Thomas Büttner, Guido Fechner, Jan-Frederic Lau, Philipp Krausewitz

**Affiliations:** Klinik und Poliklinik für Urologie und Kinderurologie, Uniklinik Bonn, Venusberg-Campus 1, 53127 Bonn, Deutschland

**Keywords:** Harnblasenamyloidose, Dysurie, Transurethrale Resektion der Harnblase, Urothelkarzinom, Makrohämaturie, Bladder amyloidosis, Dysuria, Transurethral resection of the bladder, Urothelial carcinoma, Hematuria

## Abstract

Die isolierte Amyloidose der Harnblase ist eine durch Amyloidablagerungen charakterisierte seltene Erkrankung. Wir berichten über einen Patienten mit rezidivierender schmerzloser Makrohämaturie sowie irritativen und dysurischen Miktionsbeschwerden – charakteristischen Symptomen der Harnblasenamyloidose. Der Fallbericht diskutiert die Bedeutung verschiedener Diagnose- und Therapiemethoden, Intervallen und Techniken der Verlaufskontrolle sowie Maßnahmen der langfristigen Symptomkontrolle.

## Abstract (en)

Isolated bladder amyloidosis is a rare condition characterized by amyloid deposits within the bladder wall. We present the case of a patient with recurrent, painless gross hematuria accompanied by irritative and dysuric voiding symptoms—clinical features typical of bladder amyloidosis. This case highlights the diagnostic challenges and therapeutic strategies associated with the disease, emphasizing the importance of appropriate surveillance intervals, follow-up techniques, and long-term symptom management.

## Anamnese

Ein 49-jähriger Patient stellte sich 2009 mit Dysurie, Makrohämaturie und Unterbauchschmerzen vor. Ohne urologische Vorerkrankungen oder Nikotinabusus erhielt er initial eine antibiotische Therapie bei Verdacht auf Harnwegsinfekt. Bis auf eine arterielle Hypertonie lagen keine Vorerkrankungen vor; zudem bestanden weder Allergien noch eine antikoagulative Medikation.

## Befund und Diagnose

**Abb. 1:**
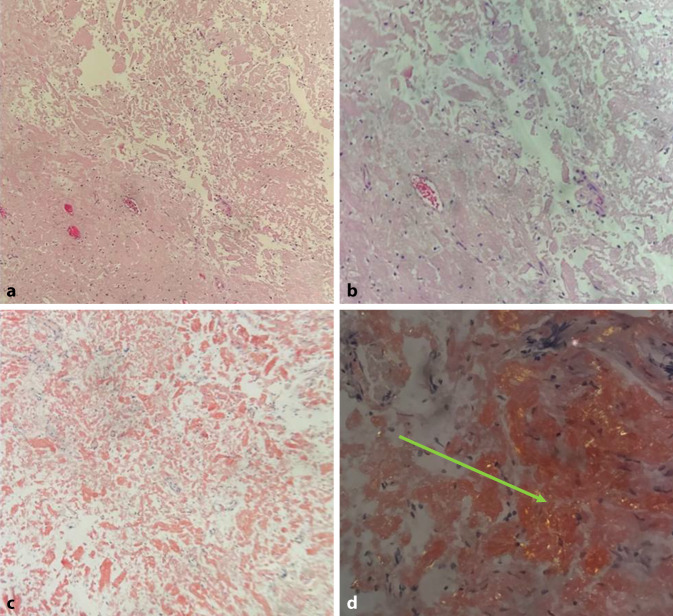
**a** In amorpher Substanz gelagerte Fibrozyten (HE (Hämatoxylin-Eosin)). **b** Ausschnitt aus **a** in höherer Vergrößerung (HE). **c** Kongorot-Färbung: rötliche amorphe Substanz. **d** Amyloidnachweis polarisationsoptisch: grüne Doppelbrechung (siehe Pfeil auf Amyloid gerichtet)

Nach initial klinischer Besserung unter Antibiose erfolgte bei rezidivierender Makrohämaturie die Sonographie des Urogenitaltraktes sowie ein Ausscheidungsurogramm ohne Auffälligkeiten. Zystoskopisch zeigte sich ein papillärer Harnblasentumor (~1 cm). Bei Malignitätsverdacht erfolgte die transurethrale Resektion der Harnblase (TURB). Histologisch ergab sich keine Malignität bei Urozystitisnachweis. Bei wiederkehrender schmerzloser Makrohämaturie wurde 2014 erneut eine TURB durchgeführt und histologisch die Diagnose „Amyloidose der Harnblase“ gestellt (Abb. 1: mittels Kongorot-Färbung nachgewiesene typische Proteinablagerungen). Intraoperativ zeigte sich diesmal eine multifokal zystitisch gerötete Mukosa am Blasenhals sowie wenige Millimeter messende blutgefüllte papilläre Strukturen der Seitenwände.

## Therapie und Verlauf

In 2015–2018 führten wir jährliche Magnetresosnanztomografien(MRT)-Beckenaufnahmen und Urinzytologien zwecks Rezidivausschluss der Amyloidose und Malignomausschluss ohne Auffälligkeiten durch. Bis 2023 erfolgten bei Makrohämaturie insgesamt 6 TURB zur Symptomkontrolle. Hier zeigte sich histologisch bei wiederholt endoskopisch multifokalen, blutig belegten grobblasigen Tumoren am Blasenhals und -dach eine Amyloidose der Harnblase ohne Malignität. 2019 wurde 4 Jahre nach Erstdiagnose eine systemische Amyloidose abgeklärt und ausgeschlossen. Zuletzt empfahlen wir keine Routinediagnostik und die Wiedervorstellung bei erneuter Symptomatik.

## Diskussion

Die isolierte Amyloidose der Harnblase ist auf die pathologische Ablagerung fehlgefalteter Amyloidproteine (Abb. 1) zurückzuführen [9]. Insgesamt sind über 20 Subtypen beschrieben. Die Erkrankung kommt sporadisch oder auch familiär gehäuft als systemische Erkrankung oder lokalisiert vor. Die Inzidenz der primären, systemischen Amyloidose beträgt ca. 1/100.000/Jahr, die sekundäre, isolierte bzw. organbegrenzte Form ist deutlich seltener [10]. Die Harnblase ist im Vergleich z. B. zur Niere („Plasmozytomniere“) bei systemischer Amyloidose nur selten betroffen [10]. Neben der Harnblase können auch Prostata, Urethra und Ureteren betroffen sein [11]. Allerdings scheint die Harnblasenamyloidose dennoch häufig Ort der Erstmanifestation einer systemischen Amyloidose zu sein [12].

Im Durchschnitt sind Patienten mit Harnblasenamyloidose 60 Jahre alt (Vergleich Urothelkarzinom: 74 Jahre), männlich (zwei Drittel der Fälle) und weisen unifokale intravesikale Läsionen auf (60 %; [7, 8]). Die Klinik ist heterogen mit zystitisassoziierten Beschwerden und Makrohämaturie [2, 7, 8]. In 30 % der Fälle kommt es zum Rezidiv innerhalb von 5 Jahren (81 % nach 2 Jahren, davon 45 % im ersten Jahr; [5]). Unser Patient entspricht in einigen Punkten dem „typischen“ Bild (Klinik, männlich, hohe Rezidivrate), wies jedoch multifokale Tumoren auf und war deutlich jünger.

Aufgrund der häufigen Überschneidungen in Bezug auf Alter und Klinik muss die Harnblasenamyloidose gegen das Urothelkarzinom und Zystitis abgegrenzt werden [2, 4, 5, 7]. Wie im aktuellen Fallbericht wird in der Literatur eine Symptomlinderung durch antibiotische Therapie beschrieben. Yu et al. diskutieren in diesem Zusammenhang die Genese der Amyloidablagerungen als Folge chronisch entzündlicher Prozesse [8]. Hinsichtlich etwaiger Überschneidungen mit dem Urothelkarzinom beschreiben Pyrgidis et al. in der bis dato größten Serie mit 184 Patienten ein Tumornachweis in bis zu 11 % der Fälle, wobei 9 % simultan vorlagen und 2 % im Verlauf diagnostiziert wurden [5]. Inwiefern eine maligne Transformation von vesikalen Amyloidosebefunden beachtet werden muss, bleibt somit unklar [5]. Chronisch entzündliche Prozesse müssen jedoch sowohl als möglicher Auslöser der Amyloidose als auch des Urothelkarzinoms bedacht werden. Vor diesem Hintergrund sollte auch eine frühzeitige TURB asymptomatischer Harnblasenamyloidosebefunde erwogen werden. Insbesondere, da der endoskopische Befund keine Abgrenzung zwischen malignen und benignen Läsionen erlaubt. Die Durchführung einer Urinzytologie kann hier hilfreich sein, wobei sie keinen Hinweis auf eine Amyloidose gibt [8]. Letztlich wird auf Basis spärlicher Literatur bei zystoskopischem Verdacht und oder Symptomatik eine TURB zur diagnostischen Sicherung und simultanen Therapie der Harnblasenamyloidose angeraten [2, 4, 5, 8]. Zur Reduktion der Rezidivfrequenz sind adjuvante Maßnahmen nach TURB mittels oralem Colchizin oder Dimethylsulfat-Instillationen (DMSO) beschrieben. Diese Therapien haben jedoch keine Zulassung für diese Indikation, worüber die Patienten aufgeklärt werden müssen. Eine solche adjuvante Therapie sollte v. a. bei diffusem Befall und inkompletter Resektion der Befunde in Betracht gezogen werden, wobei der Wirkungsgrad dieser Off-label-Nutzung unklar bleibt [3–5, 8].

Zur Verlaufskontrolle der Harnblasenamyloidose werden regelmäßige Zystoskopien als auch schnittbildgebende Verfahren diskutiert. Allerdings können weder Computertomographie noch MRT (inklusive multiparametrisches MRT [6]) eine Amyloidose sicher diagnostizieren noch gegen maligne Blasentumoren abgrenzen [1, 4, 5, 8]. Auch Biomarker existieren nicht. Bei hoher Rezidivrate sowie der Gefahr der malignen Transformation wurden bislang sonographische und zystoskopische Verlaufsuntersuchungen in regelmäßigen Intervallen nach Erstdiagnose nach 3, 12 und 24 Monaten mit anschließenden symptomorientieren Kontrollen empfohlen [4, 5, 8]. Bei fehlender Symptomkontrolle, ausgeprägten Befunden oder häufigen Rezidiven sind zudem als Ultima Ratio Zystektomien beschrieben [2, 5, 8]. Dabei ist eine frühzeitige Erkennung und Behandlung potenziell komplikativer systemischer Veränderungen (Neuropathie, Kardiomyopathie) essentiell. Entgegen unserem Fallbeispiel sollte nach Diagnosestellung mittels TURB immer auch eine systemischen Amyloidose eruiert werden. Neben einer internistischen wird primär eine dermatologische Vorstellung empfohlen. Therapien der systemischen Amyloidose reichen von Chemotherapien bis zur Stammzelltransplantation [10].

## Zusammenfassung und Ausblick

Dieser Fallbericht verdeutlicht Fallstricke im Zusammenhang mit der Diagnose und Therapie einer Harnblasenamyloidose. Sie kann klinisch sowohl als Tumor als auch als Harnwegsinfekt imponieren. Die TURB ist Diagnostik und Therapie der Wahl. Rezidive sind in einem Drittel der Fälle zu erwarten. CT und MRT haben in der Verlaufskontrolle derzeit keinen Stellenwert. Unklar bleibt, ob Kontrollzystoskopien terminiert oder symptomorientiert durchgeführt werden sollten. Bei rarer Datenlage entschieden wir uns damals für regelmäßige schnittbildgebende und zystoskopische Kontrollen. In Anbetracht aktueller Erkenntnisse, insbesondere bei geringem Risiko der malignen Transformation, ist aus unserer Sicht ein symptomorientiertes Vorgehen mittels Zystoskopie und TURB ohne weitere Schnittbildgebung oder medikamentöse Therapien im Follow-up der isolierten Harnblasenamyloidose zu empfehlen. Bei schweren Verläufen oder häufigen Rezidiven können zudem adjuvante Therapien im „off label use“ erwogen werden. Die Ultima Ratio der Zystektomie ist nur in Ausnahmefällen erforderlich. Neben der lokalen Behandlung ist die Notwendigkeit der frühzeitigen Abklärung einer systemischen Amyloidose nach Erstdiagnose einer Harnblasenamyloidose hervorzuheben.

## References

[1] Kato H, Toei H, Furuse M, Suzuki K, Hironaka M, Saito K (2003) Primary localized amyloidosis of the urinary bladder. Eur Radiol 13(6):L109–L112 16440218 10.1007/s00330-002-1793-4

[2] Kinzel RC, Harrison EG, Utz DC (1961) Primary localized amyloidosis of the bladder. J Urol 85:785–795 13756175 10.1016/S0022-5347(17)65426-0

[3] Malek RS, Wahner-Roedler DL, Gertz MA, Kyle RA (2002) Primary localized amyloidosis of the bladder: experience with dimethyl sulfoxide therapy. J Urol 168(3):1018–1020 12187212 10.1016/S0022-5347(05)64564-8

[4] Merrimen JLO, Alkhudair WK, Gupta R (2006) Localized amyloidosis of the urinary tract: case series of nine patients. Urology 67(5):904–909 16635518 10.1016/j.urology.2005.11.029

[5] Pyrgidis N, Mykoniatis I, Pegios VF, Sokolakis I, Hatzichristodoulou G, Bourdoumis A et al (2021) Amyloidosis of the Urinary Bladder: A Systematic Review and a Proposed Management Algorithm. Urology 156:e12–e19 34314752 10.1016/j.urology.2021.07.013

[6] Rayn KN, Hale GR, Bloom JB, Gold SA, Carvalho FLF, Mehralivand S et al (2018) Incidental bladder cancers found on multiparametric MRI of the prostate gland: a single center experience. Diagn Interv Radiol 24(5):316–320 30211685 10.5152/dir.2018.18102 PMC6135055

[7] Tirzaman O, Wahner-Roedler DL, Malek RS, Sebo TJ, Li CY, Kyle RA (2000) Primary localized amyloidosis of the urinary bladder: a case series of 31 patients. Mayo Clin Proc 75(12):1264–1268 11126834 10.4065/75.12.1264

[8] Yu Z, Yan L, Wang H, Hang G, Wang Y, Wen Q, Chen B (2022) Bladder triangle amyloidosis: A case report and literature review. Medicine 101(49):e32179 36626417 10.1097/MD.0000000000032179 PMC9750701

[9] Böcker W, Denk H, Heitz PU (2012) Pathologie

[10] Arasteh K (2018) Duale Reihe Innere Medizin 4. Überarbeitete Auflage

[11] Zimmer G, Weber G, Braun HP, Bersch W (1989) Bericht über zwei Fälle einer isolierten, primären Amyloidose der männlichen Harnröhre und der Harnblase einer Frau. Urol Ausg A 28(6):363–366 2603282

[12] Gilani SI, Dasari S, Tekin B, Hernandez LH, Cheville JC, Jimenez RE (2023) Identification of amyloidosis of the urinary tract and prostate: Opportunities for early diagnosis & intervention in systematic disease. Hum Pathol 142:62–67 37979953 10.1016/j.humpath.2023.11.001

